# Peer mentoring in psychiatry: a trainee-led initiative

**DOI:** 10.1192/bjo.2021.418

**Published:** 2021-06-18

**Authors:** Zoe Moore, Linda Irwin, Stuart Brown, Julie Anderson, Stephen Moore

**Affiliations:** 1 Belfast Health and Social CareTrust; 2 Northern Health and Social Care Trust; 3 South Eastern Health and Social Care Trust

## Abstract

**Aims:**

Our aim was to establish a Peer Mentoring Network within Psychiatry Training in Northern Ireland.

Recognising that starting a new job can be a stressful time in any junior doctor's career, we wanted to ensure that new Core Trainees (CT1s) joining our Specialty Programme were well supported through this transition.

Although Clinical and Educational Supervision is well established in providing a support structure for trainees, we believed that a peer mentoring relationship, (with allocation of a Higher Psychiatry Trainee as mentor), would be of additional benefit.

It was hoped that the scheme would prove mutually beneficial to both mentee and mentor.

**Method:**

We delivered a presentation at CT1 induction and sent out follow-up emails to encourage participation. Higher trainees were also sent information via email and asked to complete a basic application form if interested in becoming a mentor. Prospective mentors then attended a one-day training session.

Two lead mentors, (also higher trainees), were allocated to oversee the scheme, with additional supervision from two lead Consultants. Mentor-Mentee matches were made based on information such as location, sub-specialty affiliations and outside interests.

Matched pairs were advised about the intended frequency and nature of contacts. Check-in emails were sent halfway through the year and feedback evaluations completed at the end.

**Result:**

95% of trainees who completed the evaluations said they would recommend the scheme to colleagues.

Mentees reported benefits in terms of personal and professional development, whilst mentors reported improved listening, coaching, and supervisory skills.

A small number of trainees highlighted that 6 monthly rotations impacted on ability to maintain face to face contacts.

Recruitment and engagement have improved annually. We are currently running the third year of the scheme and have achieved 100% uptake amongst CT1s and are over-subscribed with mentors, (19 mentors to 13 mentees).

**Conclusion:**

The majority of feedback received has been positive and interest in the scheme continues to grow.

Potential issues relating to location of postings has been overcome, at least in part, by recent changes to ways of working and the use of alternative forms of contact, such as video calling.

Having exceeded demand in terms of mentor recruitment, we hope to extend the scheme to include trainees of other grades, and particularly those who are new to Northern Ireland.

We are excited to see where the next stage of our journey takes us and hope that others will be inspired to embark on similar schemes within their areas of work.

